# Serum response factor modulates neuron survival during peripheral axon injury

**DOI:** 10.1186/1742-2094-9-78

**Published:** 2012-04-26

**Authors:** Sina Stern, Daniela Sinske, Bernd Knöll

**Affiliations:** 1 Department Molecular Biology, Interfaculty Institute for Cell Biology, Eberhard Karls University Tübingen, Auf der Morgenstelle 15, Tübingen, 72076, Germany; 2 Current addresses: Institute for Physiological Chemistry, Ulm University, Ulm, 89081, Germany; 3 Deutsches Zentrum für Neurodegenerative Erkrankungen (DZNE), Bonn, 53175, Germany

**Keywords:** Facial nerve, Immune cell, Motorneuron, Regeneration, SRF, Axon, Microglia

## Abstract

**Background:**

The transcription factor SRF (serum response factor) mediates neuronal survival *in vitro*. However, data available so far suggest that SRF is largely dispensable for neuron survival during physiological brain function.

**Findings:**

Here, we demonstrate that upon neuronal injury, that is facial nerve transection, constitutively-active SRF-VP16 enhances motorneuron survival. SRF-VP16 suppressed active caspase 3 abundance *in vitro* and enhanced neuron survival upon camptothecin induced apoptosis. Following nerve fiber injury *in vitro*, SRF-VP16 improved survival of neurons and re-growth of severed neurites. Further, SRF-VP16 enhanced immune responses (that is microglia and T cell activation) associated with neuronal injury *in vivo.* Genome-wide transcriptomics identified target genes associated with axonal injury and modulated by SRF-VP16.

**Conclusion:**

In sum, this is a first report describing a neuronal injury-related survival function for SRF.

## Background

The gene regulator SRF modulates multiple aspects of neuronal motility. In SRF-deficient mice, cell migration, neurite outgrowth, branching, growth cone shape and axon guidance are impaired. In turn, constitutively-active SRF-VP16, a fusion protein of SRF and the viral VP16 transactivation domain, enhances neuronal motility [1]. Thus, SRF’s impact on physiological neuronal motility might proof beneficial also during axonal regeneration, that is the stimulation of regrowth of severed nerve fibers.

In addition to cell differentiation, SRF has been implicated in cell survival of various cell types including hepatocytes [2], thymocytes [3], heart cells [4], and during embryogenesis [5]. In embryonic stem cells lacking SRF apoptosis was strongly upregulated [4]. The latter result is in line with downregulation of the antiapoptotic protein Bcl-2 upon SRF-deficiency. *Bcl-2* was identified as SRF target gene in the same study [5]. In primary cortical neurons, SRF overexpression mediates BDNF-dependent cell survival in various paradigms of neuronal injury [6]. Also, SRF conveys expression of the immediate early gene (IEG) Cyr61 during neuronal cell death [7].

SRF operates through interaction with co-factors of the MRTF (myocardin-related transcription factors) and TCF (ternary complex factors) family. Through interaction with TCFs SRF can mediate an IEG response of for example *c-fos, Egr1* and *Arc.* IEGs are well-established molecular switches of cell survival *vs.* cell death [8]. Further, while interacting with MRTFs SRF directs expression of actin isoforms (*Acta, Actb, Actc*) or actin-binding proteins (for example tropomyosin, calponin and gelsolin) thereby regulating cytoskeletal dynamics [1,9].

Similar to SRF, MRTF-A and the TCF Elk-1 enhance cell survival of primary neurons [6,10-13] and non-neuronal cells [14]. In opposite to primary neurons, cell survival and apoptosis are not overtly altered during physiological nervous system development as revealed by SRF-deficient mice [15-17]. Indeed, apoptosis was only elevated in the subventricular zone of SRF-deficient mice [15] but not documented in for example cortical, hippocampal, striatal and peripheral neurons [15-17]. This suggests that SRF is not a major neuronal survival regulator during physiological brain development.

As mentioned above, SRF and co-factors mediate injury-related neuronal survival *in vitro*. Thus, an *in vivo* function of SRF in neuronal survival (which has not been demonstrated so far) might become apparent during application of neuronal injury.

Here we applied facial nerve injury in adult mice to investigate a role of SRF-VP16 in survival of facial motorneurons *in vivo*. In mice, the bilateral facial nerve innervates muscles regulating whisker pad and eyelid movements, for example [18]. Facial nerve axotomy is a model system for studying motorneuron survival, axonal regeneration as well as neuron and immune cell interactions during neuronal injury. We observed an SRF-VP16 dependent increase in motorneuron survival *in vivo*. In addition*,* SRF-VP16 enhanced outgrowth and survival of transected primary neurons *in vitro*. Mechanistically this SRF-VP16 function involves suppression of active caspase 3 expression *in vitro* and increased microglia and T cell activation around transected motorneurons *in vivo*. Finally, using transcriptomics, we provide axonal injury-induced and SRF-VP16 modulated target genes potentially associated with neuronal survival.

## Methods

### Facial nerve transection

The facial nerve transection was performed as described in [19]. Adult wild-type mice (>2 month) were anaesthetized, a skin incision was made behind the left ear and the facial nerve was exposed. In experiments with no virus application, the nerve was transected with small microscissors about 2 mm posterior to the foramen stylomastoideum. For viral infection, 1 μl virus was injected into the facial nerve using a 26 G Hamilton syringe. Afterwards, the nerve was transected and another 1 μl of virus was injected into the nerve stump. Of note, this virus injection with a syringe causes already a facial nerve lesion. Therefore it is only possible to delineate SRF-VP16 specific effects on the basis of experiments employing control virus, SRF-∆MADS-VP16. Cesium-chloride purified SRF-VP16 (4.6 × 10^12^ PFU/mL) and SRF-∆MADS-VP16 (4.9 × 10^12^ PFU/mL) adenoviral particles were purchased from Vector Biolabs. Both viruses drive GFP expression via a second CMV promoter. Absence of eyelid closure and whisker movement ensured successful nerve transection. All experiments are in accordance with institutional regulations by the local animal ethical committee (Regierungspräsidium Tübingen).

### Histology

Brains were fixed in 4% PFA/PBS overnight followed by preparation of 60 μm vibratome slices. Immunohistochemistry was performed using Biotin-conjugated secondary antibodies (1:500; Vector) and peroxidase-based detection systems using the ABC complex (Vector) and DAB as substrate. Primary antibodies included anti-IBA1 (rabbit, 1:500; Wako) and anti-CD3 (mouse, 1:1,000; Dr. G. Jung, Tübingen University).

### Cell biology

Primary neurons were prepared as before [20]. Hippocampal neurons derived from wild-type or SRF-deficient mice [15] were electroporated with SRF-VP16 or SRF-ΔMADS-VP16 and cultured for 72 h. Neurons were electroporated with 3 μg of the plasmids using Amaxa nucleofection resulting on average in 30% to 40% transfected cells. Neurons were stimulated for 1 h with myelin (12 μg/ml). Protein lysates were prepared as before [21]. Rabbit anti-active caspase 3 (Cell Signaling; 1:1,000) and mouse anti-GAPDH (Acris; 1:50,000) antibodies were used.

For neuronal injury experiments *in vitro*, hippocampal neurons were grown on poly-L-lysine and laminin coated video dishes. One neurite/neuron was cut with a micro-scalpel driven by an InjectMan® NI 2 Micromanipulator (Eppendorf). The cell reaction was monitored in a life cell imaging set-up (37°C, 5% CO_2_; Zeiss, Axiovert 200 M) every 5 min for a total of 6 h. Ten neurons/condition in 13 independent experiments were evaluated.

Neurons were infected with 1 × 10^8^ PFU/ml adenoviral particles expressing GFP alone, SRF-ΔMADS-VP16:GFP or SRF-VP16:GFP 5 h after plating. The next day, cultures were treated overnight (17 h) with camptothecin at 0.1, 1, or 3 μΜ followed by immunocytochemistry.

### Immunocytochemistry

Cells were fixed for 15 min in 4% PFA/5% Sucrose/PBS, permeabilized for 5 min in 0.1% Triton-X-100/PBS and blocked for 30 min in 2% BSA/PBS. Primary antibodies were incubated for 2 h at room temperature as follows: rabbit anti-active caspase 3 (Cell Signaling; 1:750; #6991), mouse anti-GFP (Roche; 1:1,000). First antibodies were detected with Alexa 488, or 546 conjugated secondary antibodies (1:1,000; Molecular Probes), followed by DAPI-staining.

### Microarrays

The facial nuclei were dissected from 300 μm brainstem sections prepared with a tissue chopper using tungsten needles. Facial nuclei of four mice/ condition were pooled and resulted on average between 0.5 and 1 μg RNA. Total RNA was isolated with the RNeasy kit (Qiagen). RNA of 0.1 μg was processed on Affymetrix GeneChips (Mouse Gene 1.0 ST array) according to protocols of the Microarray Facility Tübingen (http://www.microarray-facility.com/cms/index.php). Raw data normalized to the control sample were analyzed in such way that only genes with a fold-change of ≥ 1.5 (up- or down-regulated) were carried forward. Genes were considered SRF-VP16 specific if their fold-change differed two-fold from the respective factor obtained for SRF-ΔMADS-VP16.

### Quantitative real-time PCR (qPCR)

Total RNA derived from facial nuclei of four animals was isolated with the RNeasy kit (Qiagen). Reverse transcription was performed with 0.5 to 1 μg RNA using reverse transcriptase (Promega) and random hexamers. qPCR was performed on ABI PRISM 7700 Sequence Detector with the Power PCR SYBR green PCR master mix (Applied Biosystems). Expression was determined in relation to *Gapdh* RNA levels. Mouse primers used were as follows: *Cnn1* (fwd: GAA GGT CAA TGA GTC AAC TCA GAA; rev: CCA TAC TTG GTA ATG GCT TTG A), Sprr1a (fwd: CCT GCT CTT CTC TGA GTA TTA GGA C; rev: GCT GCT TCA CCT GCT GCT), *Atf3* (fwd: GCT GGA GTC AGT TAC CGT CAA; rev: CGC CTC CTT TTC CTC TCA T), *Gpr15*1 (fwd: TGA CGT GGA GCA GTT TTG G; rev: GGG TCA TTG TCT TGT GCT GA), *Gal* (fwd: CAG TTT CTT GCA CCT TAA AGA GG; rev: GGT CTC AGG ACT TCT CTA GGT CTT C), *Npy* (fwd: AGA AAA CGC CCC CAG AAC; rev: GAT GAG GGT GGA AAC TTG GA), *Sox11* (fwd: GAG CTG AGC GAG ATG ATC G; rev: GAA CAC CAG GTC GGA GAA GT), *Srf* (fwd: TGT GCA GGC CAT TCA TGT G; rev: ACA GAC GAC GTC ATG ATG GTG), *Egr1* (fwd: GCC GAG CGA ACA ACC CTA T; rev: TCC ACC ATC GCC TTC TCA TT), *Actn3* (fwd: ACCACTTTGACCGGAAGCG; rev: GGAGATGAGACAAGCTCGGAA), *Acta2* (fwd: CAG CAA ACA GGA ATA CGA CGA A; rev: TGT GTG CTA GAG GCA GAG CAG).

### Statistics and quantification

Numbers of independent experiments or animals are indicated in figure bars. For all cell culture experiments at least three independent cultures derived from different animals were analyzed. For quantification of neuron numbers in facial nerve injury experiments, all sections (that is 10 to 15 sections/animal) were evaluated. Neurons were scored as non-degenerated, if they protruded at least one neurite and if the cell body showed a typical angled shape. For microglia and T cell numbers 4 to 6 sections/ animal were analyzed. Statistical significance was calculated using two-tailed t test or, where appropriate, a one-way analysis of variance (ANOVA) with a Bonferroni post hoc test.

*, **, and *** indicates *P* ≤ 0.05, 0.01, and 0.001, respectively. Standard deviation is provided if not mentioned otherwise.

## Results

### SRF-VP16 enhances motorneuron survival *in vivo*

**Figure 1:**
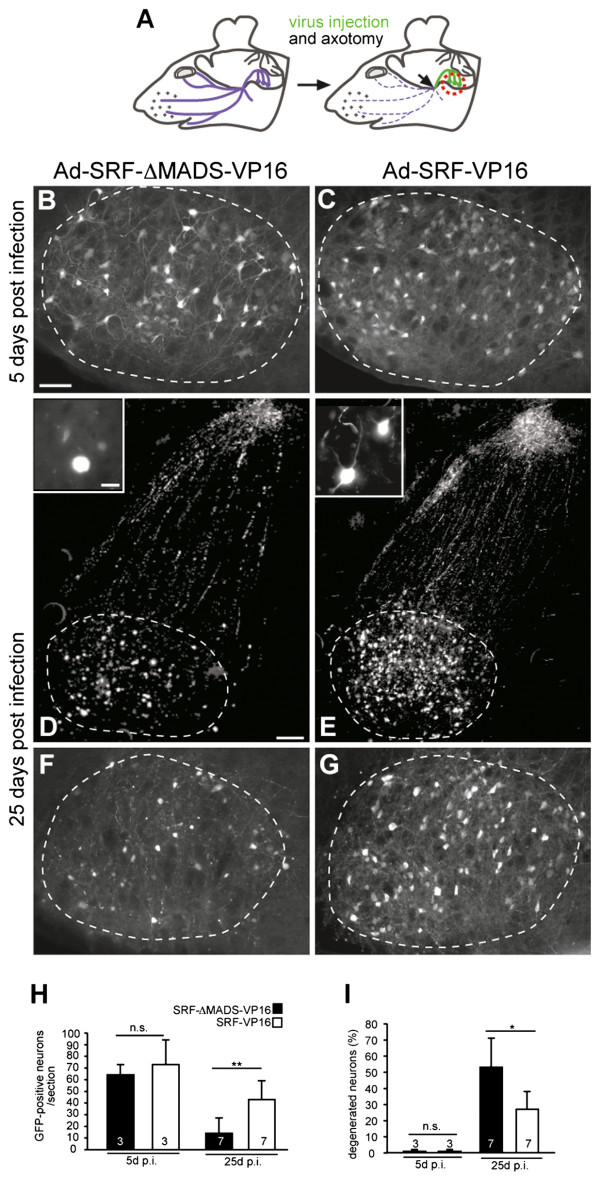
**SRF-VP16 enhances survival of facial motorneurons.** (**A**) (left) The facial nerve is outlined in blue. (right) Virus injection (green) and position of axotomy is depicted (arrow). Pictures in (**B-G**) were taken from the facial nucleus whose position is indicated by the red circle. Facial motorneurons express either SRF-VP16 or SRF-ΔMADS-VP16 along with GFP, whose expression is depicted in (B-G). (**B**, **C**) Facial nuclei of an SRF-ΔMADS-VP16 (B) or SRF-VP16 (C) expressing animal taken at 5 days post infection (d.p.i.) and lesion. No obvious differences were discernable. (**D**-G) The facial nucleus of an SRF-ΔMADS-VP16 (D, F) or SRF-VP16 (**E**, **G**) infected animal at 25 d.p.i/lesion. In SRF-ΔMADS-VP16 (D, F) compared to SRF-VP16 (E, G) numbers of surviving neurons are reduced. SRF-ΔMADS-VP16 expressing neurons are atrophic and assume a “bleb-like” morphology without innervation (see insert in **D**). SRF-VP16 neurons protrude neurites and cell bodies are squared in shape (insert in E). (**H**) Numbers of GFP-positive neurons/section are indicated. (**I**) At 25 d.p.i., but not 5 d.p.i. SRF-ΔMADS-VP16, in contrast to SRF-VP16 expressing neurons were degenerated. Dashed lines depict outlines of the facial nuclei. Scale-bar (B-G) = 100 μm; inserts = 10 μm.

To investigate a role of SRF in neuron survival, we employed a well-established model system of neuronal injury, that is unilateral de-afferentiation of facial motorneurons in mice (Figure 1A). The transected facial nerve was infected with viral particles expressing GFP in addition to SRF-ΔMADS-VP16 or SRF-VP16. SRF-VP16 consists of SRF fused to the viral VP16 transactivation domain. To control for VP16 off-target effects, SRF-ΔMADS-VP16, lacking DNA binding activity, was used as control [20]. SRF expression commenced 1 day after infection. Around the virus injection site of the facial nerve, SRF was also found in fibroblasts and glial cells, whereas in the facial nucleus - after retrograde viral transport - SRF expression was motorneuron restricted (Figure 1Additional file 1: Movie S1 and Additional file 2: Movie S2 and data not shown).

Survival was quantified by analyzing number and morphology of GFP-positive motorneurons in the facial nucleus. SRF-VP16 increased the number of surviving motorneurons compared to SRF-ΔMADS-VP16 (Figure 1, Additional file 1: Movie S1 and Additional file 2: Movie S2). At 5 days post viral infection (d.p.i.) and axotomy, the number of GFP-positive neurons expressing SRF-VP16 or SRF-ΔMADS-VP16 was comparable (Figure 1B, 1C, and 1H). However, at 25 d.p.i., numbers of surviving SRF-VP16 positive neurons after transection exceeded those expressing SRF-ΔMADS-VP16 about three-fold (Figure 1D-H).

We also inspected motorneuron morphology. SRF-VP16 expressing neurons appeared less degenerated as assessed by two parameters: neurite innervation and shrunk atrophic cell bodies. At 5 d.p.i, SRF-VP16 and SRF-ΔMADS-VP16 expressing neurons did not differ with regard to these criteria (Figure 1B, 1C and 1I). At 25 d.p.i about 60% of SRF-ΔMADS-VP16 expressing neurons lost innervation and acquired a ‘bleb-like’ rounded-up cell morphology (Figure 1D, 1F and 1I). In contrast, SRF-VP16 suppressed neuronal degeneration, leaving only 35% of neurons atrophic (Figure 1E, 1G and 1I).

This suggests that SRF plays a role in survival of axotomized facial motorneurons.

### SRF-VP16 suppressed cell death and enhanced neurite regrowth *in vitro*

**Figure 2:**
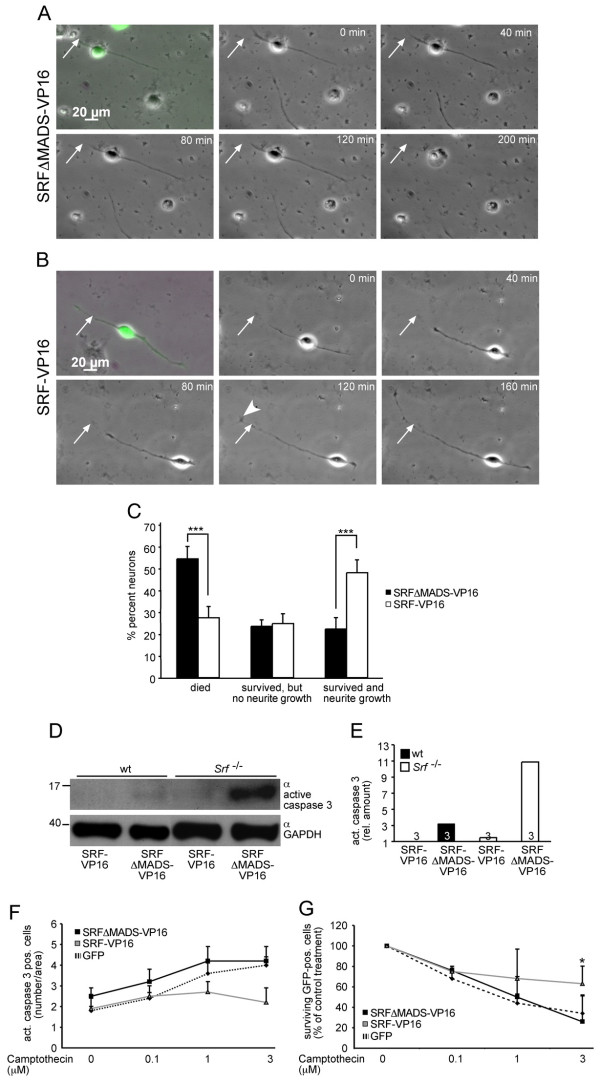
**SRF-VP16 modulates cell survival*in vitro.*** (**A**) After lesion, the neurite of an SRF-ΔMADS-VP16 expressing neuron is not re-growing and the neuron eventually dies after 200 min. The neurite was severed at the position indicated by the arrow. (**B**) A neuron expressing SRF-VP16. After transection, neurite growth is observed as well as a dynamic growth cone structure (arrowhead). Eventually at 160 min, the neurite has exceeded the original lesion position. (**C**) SRF-VP16 increased the percentage of neurons surviving nerve fiber transection and revealing re-growth of neurites. (**D**, **E**) SRF-VP16 suppressed active caspase 3 levels in wild-type and more pronounced in SRF-deficient neurons compared to neurons expressing SRF-ΔMADS-VP16. (**F**, **G**) SRF-VP16 reduced camptothecin induced neuronal cell death as quantified by counting active caspase 3 (F) or surviving GFP-positive (G) neurons.

As shown above (Figure 1), SRF-VP16 protects from motorneuron loss upon facial nerve lesion *in vivo*. To investigate whether SRF-VP16 has also an impact on neuronal survival of primary neurons we employed an *in vitro* assay of axonal injury (Figure 2). Here, neurites of primary neurons were transected using a micro-scalpel followed by recording the neuronal response with time-lapse video-microscopy (Figure 2A-C). VP16 expressing neurons were identified via GFP-expression. After lesion, neurites of an SRF-ΔMADS-VP16 expressing neuron did not re-grow and neurons frequently died (Figure 2A). In contrast, neurites of an SRF-VP16 expressing neuron were capable of re-growth, often protruded dynamic growth cones and survived neurite transection (Figure 2B; quantification in 2C). Thus, SRF-VP16 enhances neuronal survival and re-growth of severed neurites *in vitro*.

In a next step, we investigated potential mechanisms of SRF’s function in neuronal survival. SRF-VP16 might modulate motorneuron survival via blocking apoptosis. To investigate this further, we employed primary neurons assessing protein levels of the pro-apoptotic protein active caspase-3 upon SRF-VP16 expression (Figure 2D, 2E). In SRF-ΔMADS-VP16 expressing neurons, active caspase 3 levels were strongly induced. In contrast SRF-VP16 suppressed this myelin-induced activation of active caspase-3 (Figure 2D, 2E). Notably, this effect was more obvious in neurons lacking SRF compared to wild-type neurons (Figure 2D and quantification in 2E).

In a further set of experiments we analyzed whether SRF-VP16 might enhance neuronal survival upon camptothecin induced DNA damage (Figure 2F2G). In control infected primary neurons either expressing GFP alone or SRF-ΔMADS-VP16, camptothecin induced apoptosis in a concentration dependent manner. This was quantified by either counting numbers of active caspase 3 positive (Figure 2F) or numbers of surviving GFP-positive neurons (Figure 2G). In contrast, SRF-VP16 expression reduced this camptothecin induced neuronal cell death compared to control constructs (Figure 2F2G). Thus, in cultures infected with adenoviral particles expressing SRF-VP16 (along with GFP) the number of active caspase 3 positive neurons was reduced (Figure 2F) whereas more GFP-positive neurons survived camptothecin treatment (Figure 2G). This finding is in agreement with previous observations made with wild-type SRF in cortical neurons [6].

Thus, results from primary neurons suggest that SRF-VP16 might down-regulate expression of pro-apoptotic proteins to enhance neuronal survival.

### SRF-VP16 modulates injury associated immune responses

**Figure 3:**
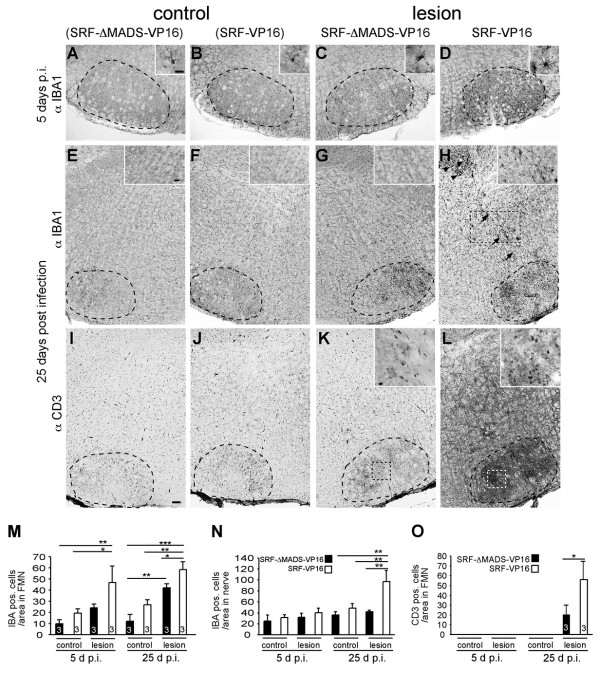
**SRF-VP16 increases microglia and T cell activation in axonal injury.** (**A-H**) Upon axotomy, microglia were activated at both time-points in the lesioned side expressing SRF-ΔMADS-VP16 compared to the control side (compare **C**, **G** with A, **E**). SRF-VP16 (**B**, **F** and **D**, **H**) enhanced microglia activation at both time-points. In addition, SRF-VP16 enhanced microglia association along the axons (arrows in H) and the nerve exit point (arrowheads in H) at 25 d.p.i. (see insert in H). (**I-L**) At 25 d.p.i., T cells entered the transected facial nucleus in control infected animals (**K**; the insert shows individual T cells), but not the intact facial nucleus (I). In animals expressing SRF-VP16, T cell infiltration was strongly enhanced in the lesion (L) but not the control side (**J**). T cells were also found along nerves (L). (**M**, **N**) Numbers of microglia/area are indicated for all conditions in the facial nucleus (M) and along the facial nerve (N). (**O**) Numbers of T cells/area are indicated for all conditions in the facial nucleus. Dashed lines depict outlines of the facial nuclei. Dashed boxes point at positions magnified by inserts. Scale-bar (A-L) = 100 μm; inserts (A-D, K, L) = 20 μm; inserts (E-H) = 100 μm.

Neuronal injury is accompanied by immune responses, for example astrocyte, microglia, and T cell activation and their subsequent infiltration of lesioned neuronal tissue. Injury-related immune responses might dampen as well as exacerbate neuronal loss [18,22]. Regarding the facial nerve lesion model, peri-neuronal accumulation of microglia cells facilitates axonal regeneration [23]. Also T cells were assigned important roles for the immune surveillance during facial nerve injury [24]. Given this important link between an immune response and neuronal injury, we asked whether SRF-VP16 might modulate immune responses associated with axon injury (Figure 3).

Firstly, we inspected microglia activation in the de-afferented facial nucleus. Microglia were expectedly elevated at the lesion side compared to the control side at 5 and 25 d.p.i (Figure 3A, 3E and 3C, 3G; 3M). SRF-VP16 augmented microglia activation in the lesioned facial nucleus at both time-points compared to SRF-ΔMADS-VP16 (Figure 3B, 3 and 3D, 3H). Notably, SRF-VP16 also enhanced microglia occupancy along the facial nerve axons and the axon exit point (arrows and arrowheads in Figure 3H, respectively; Figure 3N).

Secondly, we investigated T cells labeled with an anti-CD3 directed antibody. T cells did not enter the facial nucleus 5 d.p.i. regardless of virus type (Figure 3O). In contrast, at 25 d.p.i., we observed T cell infiltration in lesioned SRF-ΔMADS-VP16 expressing neurons but not on the uninfected control side (Figure 3I, K, and 3O). Similar to results obtained on microglia (Figure 3A-H), SRF-VP16 also enhanced T cell infiltration around motorneurons and axons (Figure 3L and 3O).

Taken together, microglia and T cell responses are augmented upon SRF-VP16 expression.

### Microarray analysis of lesion and SRF-VP16 induced transcripts

**Figure 4:**
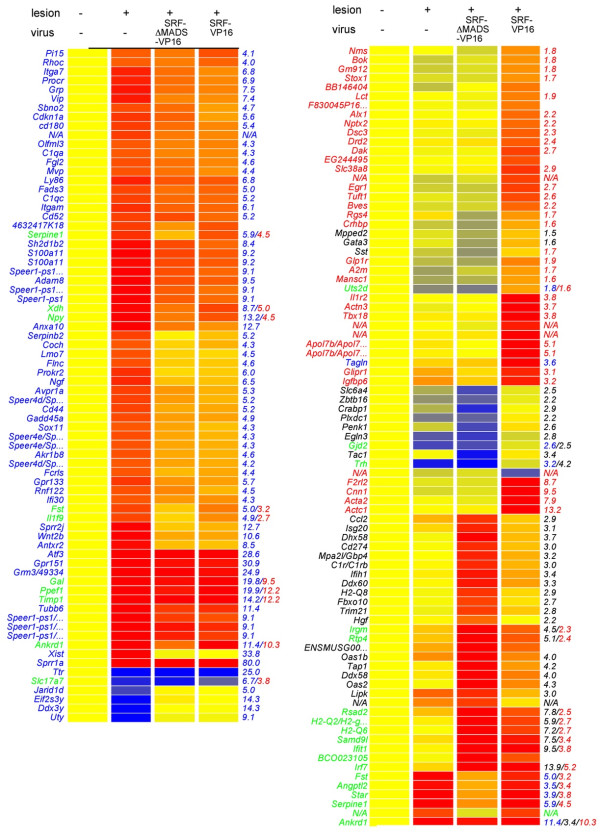
**Transcriptomics of facial nerve injury and SRF-VP16 associated genes.** Three days after facial nerve transection, facial nuclei were subjected to microarray analysis. Genes up- or down-regulated (≥ 4-fold) by facial nerve injury alone are depicted in blue. Genes specifically altered by SRF-VP16 upon nerve injury are highlighted in red. Genes in black color are modulated by SRF-ΔMADS-VP16. Genes depicted in green are modulated by lesion alone and SRF-VP16 or SRF-ΔMADS-VP16. Red colors indicate high, whereas blue colors represent low expression. All expression levels were normalized to the control condition (without lesion).

**Figure 5:**
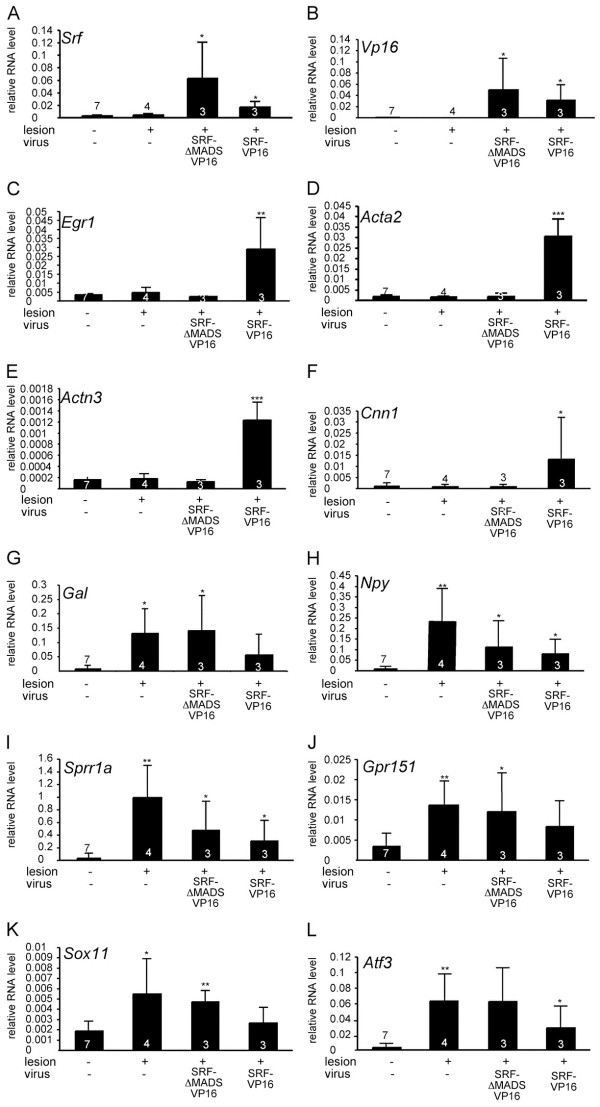
**Validation of transcriptomics data.** cDNAs derived from unlesioned, lesioned, lesioned and SRF-ΔMADS-VP16-positive and lesioned and SRF-VP16-positive facial nuclei were subjected to qPCR analysis with the indicated primers. Every bar reflects mRNA levels obtained from a cDNA sample in which facial nuclei of four independent animals were pooled. Numbers of independent cDNAs are indicated at bars. Statistical significance was calculated in relation to control (no lesion/no virus).

SRF might enhance neuronal survival through various mechanisms including regulation of survival/apoptosis related (Figure 2) and immune regulatory genes (Figure 3). To identify genes modulated by facial nerve injury *per se* and by SRF-VP16, we performed transcriptomics after three days of facial nerve lesion (Figures 4 and 5, Table 1 and Additional file 3: Table S1). For this, facial nuclei of four mice were pooled for each condition.

**Table 1:** Summary of genes most strongly regulated by lesion only or SRF-VP16

	**Lesion-specific genes**			**SRF-VP16-specific genes**		
	**Gene name**	**Gene symbol**	**Up**	**Gene name**	**Gene symbol**	**Up**
1	Small proline-rich protein 2J	Sprr2j	80.1	Actin, alpha, cardiac	Actc1	13.2
2	G protein-coupled receptor 151	Gpr151	31.0	Protein phosphatase with EF hand	Ppef1	12.2
3	Activating transcription factor 3	Atf3	28.6	Tissue inhibitor of metalloprot. 1	Timp1	12.2
4	Glutamate receptor, metab. 3	Grm3	24.9	Ankyrin repeat domain 1	Ankrd1	10.3
5	Protein phosphatase with EF	Ppef1	19.9	Calponin 1	Cnn1	9.5
6	Galanin	Gal	19.8	Coagulation factor II receptor-like 2	F2rl2	8.7
7	Tissue inhibitor of metalloprot. 1	Timp1	14.3	Actin, alpha 2, smooth muscle	Acta2	7.9
8	Neuropeptide Y	Npy	13.2	Transgelin (Sm22)	Tagln	6.4
9	Annexin a10	Anxa10	12.7	Apolipoprotein L 7b | L 7e	Apol7b/e	4.7
10	Small proline-rich protein 2 J	Sprr2j	12.7	Serine (or cysteine) peptidase inh.	Serpine1	4.5
11	Tubulin, beta 6	Tubb6	11.4	T-box18	Tbx18	3.8
12	Ankyrin repeat domain 1	Ankrd1	11.4	Interleukin 1 receptor, type II	Il1r2	3.8
13	Wingless related 2b	Wnt2b	10.6	Actinin alpha 3	Actn3	3.7
14	A disintegrin and metallopept. 8	Adam8	9.5	Angiopoietin-like 2	Angptl2	3.4
15	S100 calcium binding prot. A11	S100a11	9.2	Insulin-like growth fac. bind. prot. 6	Igfbp6	3.2
16	Xanthine dehydrogenase	Xdh	8.7	Follistatin	Fst	3.2
17	Anthrax toxin receptor 2	Antxr2	8.5	GLI pathogenesis-related 1	Glipr1	3.1
18	SH2 domain protein 1B2	Sh2d1b2	8.4	Solute carrier family 38, member 8	Slc38a8	2.9
19	Gastrin releasing peptide	Grp	7.5	Early growth response 1	Egr1	2.7
20	Vasoactive intestinal polypeptide	Vip	7.4	Dihydroxyacetone kinase 2 hom.	Dak	2.7
21	Protein C receptor, endothelial	Procr	6.9	Tuftelin 1	Tuft1	2.6
22	Integrin alpha 7	Itga7	6.8	Serum response factor	Srf	2.4
23	Lymphocyte antigen 86	Ly86	6.8	Bone marrow stromal cell antigen 1	Bst1	2.4
24	Nerve growth factor	Ngf	6.5	Dopamine receptor 2	Drd2	2.4
25	Integrin alpha M	Itgam	6.1	Desmocollin 3	Dsc3	2.3
26	Prokineticin receptor 2	Prokr2	6.0	Blood vessel epicardial substance	Bves	2.2
27	Serine (or cysteine) pept. inh.	Serpine1	5.9	ALX homeobox 1	Alx1	2.2
28	G protein-coupled receptor 133	Gpr133	5.7	Neuronal pentraxin 2	Nptx2	2.2
29	Cyclin-dep. kinase inh.1A (P21)	Cdkn1a	5.6	Glucagon-like peptide 1 receptor	Glp1r	1.9
30	GalaninCD180 antigen	Cd180	5.4	Regulator of G-protein signaling 4	Rgs4	1.7

Upon lesion only, 1,088 genes (858 up, 230 down) were regulated more than 1.5-fold compared to un-lesioned facial nuclei. Figure 4 represents those genes modulated by facial nerve injury alone regulated by a factor ≥ 4 (colored in blue). These included reported genes induced by facial nerve injury such as *Atf3GalTubb6Avpr1aVip*, and *Itga7*[18,25]. In addition, we noted that many genes encoding G-protein coupled receptors (GPCRs), hormones and small neuropeptides were modulated by facial nerve axotomy including *Avpr1aGrm3Prokr2Npr3Gpr161Gpr133Gpr84GalNpyVip*, and *Grp* (Figure 4, Table 1, and Additional file 3: Table S1).

SRF-VP16 specific genes modulated after facial nerve injury are depicted in red (Figure 4, Table 1). SRF-VP16 modulated two well-known gene sets, IEGs (for example *Egr1*, *Egr2*) and actin cytoskeletal genes (*Actc1*, *Cnn1*, *Acta2*, and *Actn3*). Similar to facial nerve injury alone (see above) we noted that several potential SRF target genes encoded components of GPCR signaling (*F2rl2*, *Glp1r*, *Rgs4*, *Crhbp*, *Nms*, *Galp*; Figure 4 and Table 1). Finally we observed genes modulated by SRF-VP16 which might link SRF activity to immune responses investigated above (Figure 1). These include tissue inhibitor of metalloproteinase (*Timp1*), interleukin receptor (*Il1r2)*, galanin-like peptide (*Galp*), neuromedin (*Nms*), and interleukin 1 (*Il1f9*) (Figure 4, Table 1 and Additional file 3: Table S1; see also discussion).

To corroborate microarray data (Figure 4), qPCR analysis of selected genes employing independent cDNA samples was performed. Indeed fold changes obtained in this qPCR analysis were comparable to the microarray data (Figure 5).

## Discussion

So far, SRF signaling was not assigned a major role in neuronal survival *in vivo*[15,16] in contrast to injury-related survival *in vitro*[6,10,12,26]. This suggests that SRF regulates neuron survival primarily in an injury related situation rather than in physiological brain development. In accordance, we here demonstrate a neuroprotective SRF-VP16 function *in vivo*, that is preventing motorneuron degeneration upon facial nucleus deafferentiation (Figure 1). Further, SRF-VP16 prevented expression of proapoptotic active caspase 3, enhanced regrowth of severed neurites *in vitro* and reduced camptothecin induced apoptosis (Figure 2). The latter might be directly linked to SRF-VP16 induced cytoskeletal genes such as actin isoforms (*Actc1Acta2),* calponin (*Cnn1*), and actinin (*Actn3*; Figure 4 and Figure 5).

### How might SRF-VP16 enhance facial motorneuron survival?

SRF-VP16 suppressed active caspase 3 *in vitro* and reduced camptothecin-induced neuronal cell death (Figure 2). This SRF-VP16 mediated reduction of active caspase 3 was stronger in primary neurons lacking SRF compared to wild-type neurons (Figure 2). Such a reduction in proapoptotic protein levels by SRF-VP16 might enhance neuronal survival also upon facial nerve injury *in vivo.* To modulate expression of apoptosis related proteins, SRF-VP16 might recruit IEGs, known regulators of neuronal survival [8], such as *Egr-1* and *Egr-2*[27] which were induced by SRF-VP16 during facial nerve lesion (Figures 4 and 5).

In contrast to primary neurons (Figure 2), we did not observe any major effect of SRF-VP16 compared to SRF-ΔMADS-VP16 on active caspase 3 and BAX expression upon facial nerve lesion of wild-type mice *in vivo* (data not shown). In addition SRF-VP16 did not alter Ki-67 expression, a proliferation marker. Ki-67 was strongly induced in lesioned facial motorneurons compared to unlesioned neurons at 7 days but notably not anymore at 21 days after lesion (data not shown).

Thus, similar to primary neurons (Figure 2), *in vivo* SRF-VP16’s potential to enhance neuronal survival might be more pronounced and only become visible in the absence of endogenous wild-type SRF. Indeed it is known that wild-type SRF competes with SRF-VP16 for access to certain SRF target gene promoters such as *Bcl-2*[8]. Here, SRF-VP16 induced *Bcl-2* mRNA levels in SRF-deficient embryonic stem cells whereas SRF-VP16 failed to induce *Bcl-2* in wild-type cells [8].

In sum, using SRF-deficient primary neurons we demonstrate that SRF-VP16 modulates apoptosis *in vitro*. Thus it will be useful to employ SRF-deficient mice to unmask SRF-VP16’s impact on apoptosis also *in vivo.*

SRF-VP16 enhanced injury associated immune responses including microglia and T cell activation (Figure 3). SRF-VP16 enhanced microglia occupancy at facial nerve axons (Figure 3). In axonal injury, immune cells such as microglia remove myelin debris and have neuroprotective potential which might enhance neuronal survival [22]. Thus, by stimulating microglia and T-cell number and infiltration in the lesioned FMN and thereby increasing motorneuron cell body and facial nerve axon occupancy with these potentially neuroprotective immune cells, SRF-VP16 might enhance neuronal survival.

Of note, SRF-VP16 expression in the facial nucleus was confined to motorneurons (Figure 1 and Additional file 1: Movie S1 and Additional file 2: Movie S2). Thus, SRF-VP16 expression in neurons might influence immune cells such as microglia and T cells via a paracrine mechanism. Such a paracrine mechanism whereby neuronal SRF affects neighboring cells via regulation of secreted molecules has been described before, for example in oligodendrocytes [28,29]. With regard to immune responses initiated upon facial nerve injury such a paracrine mechanism might involve, for example, cytokine/hormone secretion by neurons. Indeed, the genome-wide search for SRF-VP16 target genes upon nerve axotomy provides candidates (Figure 4, Table 1, and Additional file 3: Table S1). In microarray results presented in this study (Figures 4 and 5, Table 1, and Additional file 3: Table S1), facial nuclei of four animals were collected in a single biological sample. Thus, although we confirmed some results with independent cDNAs in qPCR (Figure 5), interpretation of microarray data is limited by a lack of statistical evaluation. Taking this into account, SRF target genes associated with up-regulated immune responses might include *Il1r2GalpNmsIl1f9*, and *Timp1*. For instance Timp1, a regulator of matrix metalloproteases activity and thereby modulator of, for example, microglia migration [30] is more than 12-fold induced by SRF-VP16 (Table 1).

## Conclusions

In sum, this study revealed a first neuroprotective SRF function during nervous system injury *in vivo*. SRF is involved in development and physiological function of many other organs including liver, skin, muscle, blood vessels, and, for example, the heart [9]. Thus, SRF might also be involved in survival and cellular regeneration processes of other injured organs besides the nervous system.

## Abbreviations

d.p.i, Days post infection; BDNF, Brain derived neurotrophic factor; CNS, Central nervous system; FMN, Facial motor nucleus; IEG, Immediate early gene; MRTF, Myocardin related transcription factor; PNS, Peripheral nervous system; SRF, Serum response factor; TCF, Ternary complex factor.

## Competing interest

The authors declare that they have no competing interests.

## Authors’ contributions

SS and DS performed and evaluated all experiments. BK designed the study and wrote the manuscript. All authors have read and approved the final version of the manuscript.

## Supplementary Material

Additional flie 1 **Movie S1.** GFP expression in the nucleus facialis infected with Ad-SRF-VP16. Click here for file

Additional file 2 **Movie S2.** GFP expression in the nucleus facialis infected with Ad-SRF-ΔMADS-VP16. Click here for file

Additional file 3 **Table S1.** Raw and processed data of transcriptomics. Click here for file
